# Exogenous Nucleotides Mitigate Cardiac Aging in SAMP8 Mice by Modulating Energy Metabolism Through AMPK Pathway

**DOI:** 10.3390/nu16223851

**Published:** 2024-11-11

**Authors:** Yuxiao Wu, Rui Liu, Chan Wei, Xiujuan Wang, Xin Wu, Yong Li, Meihong Xu

**Affiliations:** 1 Department of Nutrition and Food Hygiene, School of Public Health, Peking University, Beijing 100191, China; 2 Beijing Key Laboratory of Toxicological Research and Risk Assessment for Food Safety, Peking University, Beijing 100191, China

**Keywords:** exogenous nucleotides, cardiac aging, AMPK pathway, energy metabolism

## Abstract

Background: Cardiovascular disease (CVD) is the predominant cause of mortality, with aging being a significant risk factor. Nucleotides (NTs), essential for numerous biological functions, are particularly vital under conditions like aging, starvation, and nutrient deficiency. Although the antiaging benefits of exogenous NTs have been recognized in various systems, their cardiac-specific effects are not well understood. This study, therefore, investigated the impact of exogenous NTs on cardiac aging and delved into the potential mechanisms. Methods: Senescence-accelerated mouse prone-8 (SAMP8) mice were utilized, randomly assigned to one of three groups: a control group (Control), a low-dose NTs group (NTs_L), and a high-dose NTs group (NTs_H). Meanwhile, senescence-accelerated mouse resistant 1 (SAMR1) mice were set up as the SAMR1 group. Following a 9-month intervention, cardiac tissues were subjected to analysis. Results: The results showed that NTs improved the morphological structure of the cardiac tissue, enhanced the antioxidant capacity, and mitigated inflammation. Metabolomics analysis revealed that the high-dose NT intervention improved cardiac tissue energy metabolism, potentially through activating the AMPK pathway, enhanced mitochondrial biogenesis, and increased TFAM protein expression. Conclusions: Together, these results indicate that exogenous NTs exert beneficial effects on the cardiac tissues of SAMP8 mice, potentially mitigating the cardiac aging process.

## 1. Introduction

Globally, cardiovascular disease (CVD) persists as a primary cause of illness and death, with aging being a major risk factor [1,2]. The heart’s restricted capacity for self-repair leads to cumulative damage, which is linked to a diminished reserve function and a heightened risk of cardiovascular disease among the elderly [3]. Even without obvious damage, the structure and function of the heart can change with age, which appears to help increase susceptibility to heart failure in older adults [4,5]. Epidemiological evidence indicates that lifestyle factors like diet and exercise can influence age-related cardiac decline, with animal studies showing that various interventions can alter the aging process and heart failure development, suggesting potential pathways to extend cardiac health [4,6,7]. It is of great significance to pay attention to cardiac aging and take active measures to combat age-related cardiovascular diseases for improving the quality of life of the elderly.

Mitochondrial dysfunction, oxidative stress, chronic inflammation, and metabolic dysfunction are considered as the hallmarks of aging [8]. These factors interact in a complex manner to drive the aging process. Metabolic alterations, which are a common feature of cardiac aging [9], disrupt energy production and contribute to cardiomyocyte senescence. This is particularly due to mitochondrial dysfunction, which leads to an increase in reactive oxygen species (ROS) and subsequent oxidative damage [10]. Additionally, inflammatory cytokines, such as IL-6, IL-1β, and TNF-α, have been linked to cardiovascular diseases, including heart enlargement and heart failure [11]. These cytokines have been consistently associated with adverse cardiac outcomes in various studies, highlighting their role in the progression of cardiac aging [12,13,14,15].

AMP-activated protein kinase (AMPK), a central regulator of cellular energy metabolism, is associated in diverse physiological and pathological processes. The AMPK signaling pathway plays a crucial role in maintaining mitochondrial function and energy homeostasis [16]. Peroxisome proliferator-activated receptor gamma coactivator-1α (PGC-1α), abundant in cardiac tissues, is activated by AMPK to regulate mitochondrial biogenesis and energy metabolism, which are vital for heart function [17,18]. Mitochondrial transcription factor A (TFAM), the downstream target of PGC-1α, is a key regulator of mitochondrial DNA replication and transcription, which is essential for mitochondrial biogenesis and function [19]. Thus, targeting the AMPK signaling pathway may offer a potential strategy for alleviating age-related cardiac aging.

Nucleotides (NTs) are essential for various biological functions, including growth, development, metabolism, and reproduction. While the body can assimilate and use nucleotides from dietary sources, exogenous NTs are considered essential under specific circumstances, including disease, intestinal damage, immune system challenges, senescence, rapid growth phases, nutrient-deficient diets, and situations impacting endogenous synthesis and expression [20]. Persistent insufficient intake of nucleotides may result in the malfunction of multiple body systems. During the course of aging, the body’s capacity for nucleotide synthesis diminishes, necessitating the intake of exogenous nucleotides to sustain normal physiological functions. Recent research has demonstrated that exogenous nucleotides are instrumental in restoring mitochondrial function by reducing oxidative stress and promoting ATP and NAD^+^ production [21,22]. Furthermore, the effects on improving immunity and reducing the levels of inflammation were observed [21,23]. Collectively, these results suggest that exogenous NTs potentially exert anti-aging effects on cardiac aging.

Although previous studies have demonstrated the beneficial effects of exogenous NTs, their specific influence on the cardiac system remains unexplored. Therefore, this study aims to fill this gap by examining the impact of exogenous NTs on cardiac aging in senescence-accelerated mouse prone-8 (SAMP8) mice, a well-regarded model for examining the cardiovascular implications of aging [24]. The findings may provide a novel nutritional intervention strategy for the prevention and treatment of cardiac aging.

## 2. Materials and Methods

### 2.1. Materials

Zhen-Ao Biotechnology Co., Ltd. (Dalian, China) supplied the mixture of NTs, which includes 5′-guanosine monophosphate (GMP), 5′-disodium uridine monophosphate (UMP), 5′-cytidine monophosphate (CMP), and 5′-adenosine monophosphate (AMP). These nucleotides were obtained from sucrose molasses via enzymatic hydrolysis, ensuring a purity of over 99%. The proportion of components within the blend was 16:41:19:24 for 5′-AMP:5′-CMP:5′-GMPNa2:5′-UMPNa2, respectively.

### 2.2. Animals and Treatment

SAMP8 mice and SAMR1 mice (senescence-accelerated mouse resistant 1) are widely used as aging models and are well suited for the study of cardiac aging. The Laboratory Animal Center of Peking University provided 12-week-old male specific-pathogen free (SPF) SAMP8 and senescence-accelerated mouse resistant 1 (SAMR1) mice. Housed individually in barrier-grade animal room cages, they were maintained at a temperature of 24 ± 2 °C, a relative humidity between 50% and 60%, and under a 12-h light/dark cycle. The animals had ad libitum access to food and water throughout the experimental period. Following a 1-week acclimation period, 45 SAMP8 mice were randomly distributed into three groups of 15, comprising a control group (Control), a low-dose nucleotide-treated group (NTs_L), and a high-dose nucleotide-treated group (NTs_H). In parallel, 15 SAMR1 mice constituted the SAMR1 group (SAMR1). The control and SAMR1 groups received a standard diet, whereas the NT intervention groups received the same diet supplemented with 0.3 and 1.2 g/kg of NTs, respectively. The experimental setup and intervention details for the animals are summarized in Table 1. Weekly measurements and recordings of the mice’s dietary consumption and body weight were conducted, as previously detailed in our prior study [25]. After a 9-month intervention, the mice, at 12 months old, were euthanized in the morning following an 8-h fast. The hearts were then harvested and preserved at −80 °C for subsequent analysis.

### 2.3. Histomorphology Observation of the Cardiac Tissues

Cardiac tissues were fixed in 4% paraformaldehyde for 48 h and subsequently decalcified in a 15% EDTA solution at room temperature for two weeks prior to paraffin embedding. Sections were then prepared and stained with hematoxylin-eosin (HE). The microscopic examination of the cardiac tissue’s morphological structure was conducted using a BX43F microscope (Olympus, Tokyo, Japan), and relevant images were captured.

### 2.4. ELISA Analysis

For ELISA analysis, a random sample of 10 mice per group was chosen. A 10% tissue homogenate was prepared for test kit detection. The oxidative stress-associated biomarkers, including malondialdehyde (MDA), total superoxide dismutase (SOD), and glutathione peroxidase (GSH-Px), were measured using ELISA analysis. The MDA (thiobarbituric acid (TBA) method), SOD (hydroxylamine method), and GSH-Px (colorimetric method) assay kits were all purchased from the Nanjing Jiancheng Bioengineering Institute (Nanjing, Jiangsu, China).

Inflammatory biomarkers, including interleukin-1 β (IL-1β), interleukin-6 (IL-6), and tumor necrosis factor-α (TNF-α), were quantified using ELISA kits according to the protocol provided by the manufacturers (Invitrogen Co., Ltd., Waltham, MA, USA).

Mitochondrial function biomarkers, including the content of ATP, succinate dehydrogenase enzyme (SDH) activity, and citrate synthase (CS) activity, were analyzed using assay kits. The SDH activity (colorimetric method) and ATP content (colorimetric method) assay kits were both obtained from the Nanjing Jiancheng Bioengineering Institute (Nanjing, Jiangsu, China), while the CS activity assay kit was purchased from Beijing Solarbio Science & Technology Co., Ltd. (Beijing, China).

### 2.5. Western Blotting Analysis

Randomly selected Western blot analyses were performed on a subset of three mice per group. Proteins were extracted and their concentrations determined using the BCA Protein Assay Kit. The protein samples were subjected to SDS-PAGE and then transferred onto PVDF membranes. These membranes were blocked with a solution containing 5% non-fat dry milk in TBST for 1 h at room temperature. Following the blocking step, the membranes were probed with the respective primary antibodies at 4 °C over night. The primary antibodies used included anti-AMPK (mouse, 1:1000), anti-p-AMPK (Thr172) (rabbit, 1:500), anti-SIRT1 (rabbit, 1:1000), anti-PFK-2 (rabbit, 1:1000), anti-p-PFK-2 (Ser466) (rabbit, 1:1000), anti-PFK-1 (rabbit, 1:1000), anti-TFAM (rabbit, 1:2000), and anti-β-actin (mouse, 1:5000), all purchased from Immunoway (TX, USA). Anti-PGC-1α (rabbit, 1:1000) was obtained from Abcam (Cambridge, UK). On the following day, the membrane was allowed to equilibrate to room temperature before being incubated for an additional 30 min. It was then rinsed five times with TBST, each wash lasting three minutes. The goat anti-rabbit IgG (H+L) HRP, which is the secondary antibody, was prepared at a 1:10,000 dilution in 5% skim milk-TBST and incubated with the membrane under gentle agitation at room temperature for 40 min. After incubation, the membrane underwent six washes with TBST, with each wash lasting three minutes. Finally, the blots were visualized using an enhanced chemiluminescence (ECL) detection reagent, and the grayscale intensity was analyzed using ImageJ software (Total Lab Quant V11.5, Newcastle upon, Tyne, UK).

### 2.6. Metabolomics Analysis

The 600 Multiple Reaction Monitoring (600MRM) analysis (Biotree, Shanghai, China) with LC-MS/MS was conducted to analyze the cardiac tissue sample. Six mice from each group were selected at random for metabolomics analysis. A total of 1500 μL of acetonitrile-methanol-H_2_O (2:2:1, containing isotope internal standards) was added into 25 mg of each sample and was mixed with 1 bead. Samples were homogenized at 50 Hz for 4 min and were sonicated in ice-water bath for 5 min. This homogenization-sonication cycle was repeated 3 times. Subsequently, the samples were incubated at −40 °C for 2 h. Following incubation, the samples underwent centrifugation at 12,000 rpm at 4 °C for a duration of 15 min. Thereafter, 1200 μL of the supernatant from each sample was decanted into new Eppendorf tubes and dehydrated using a centrifugal evaporator. To rehydrate the samples, 120 μL of 60% acetonitrile was introduced into the Eppendorf tubes, which were then vortexed to ensure complete dissolution of the solids, followed by another centrifugation step at 12,000 rpm and 4 °C for 15 min. Ultimately, 60–70 μL of the supernatant from each sample was aliquoted into glass vials for subsequent LC-MS/MS analysis. A pool of standard metabolites served as the QC sample.

The raw data from the LC-MS/MS system were processed with SCIEX Analyst Work Station Software (1.7.2) and BIOTREE Bio Bud (2.1.4). The dataset, which encompassed peak counts, sample identifiers, and normalized peak areas, was imputed into the SIMCA16.0.2 software package (Sartorius Stedim Data Analytics AB, Umea, Sweden) for multivariate analysis including principal component analysis (PCA) and supervised orthogonal projections to latent structures-discriminate analysis (OPLS-DA). From the OPLS-DA analysis, the variable importance in the projection (VIP) values for the first principal component were extracted. Metabolites were identified as differentially expressed (DEMs) if they had a VIP > 1 and a *p*-value < 0.05 tested by a student’s *t*-test. Furthermore, pathway enrichment analysis was conducted using databases such as KEGG and MetaboAnalyst.

### 2.7. Statistical Analysis

All experimental data are expressed as mean ± standard deviation (SD). Statistical analysis was conducted using SPSS 24.0 software (IBM Corp, Armonk, NY, USA) with one-way ANOVA. In cases of equal variance, inter-group comparisons were performed using the LSD method, while the Tamhane test was applied when variances were unequal. A *p*-value < 0.05 was considered statistically significant.

## 3. Results

### 3.1. Effects of NTs on the Cardiac Tissue Morphology in SAMP8 Mice

**Figure 1:**
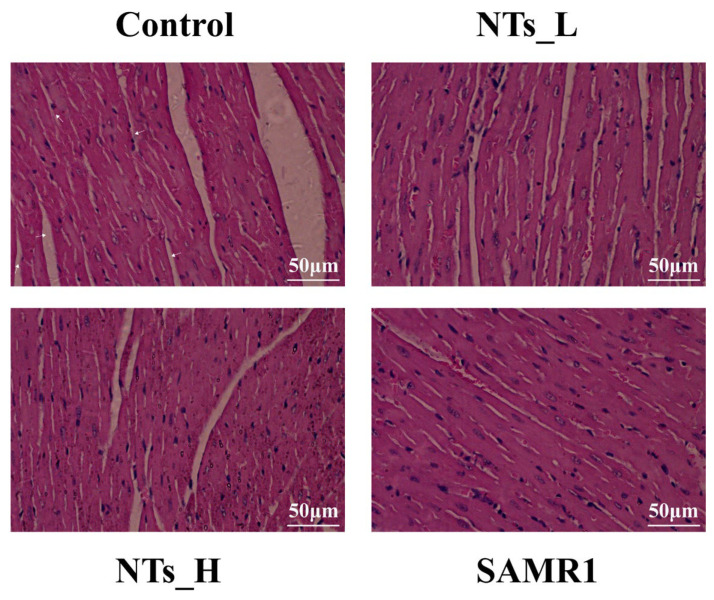
H&E staining of cardiac tissue (magnification: 400×).

The histopathological examination of cardiac tissue in mice was conducted using hematoxylin and eosin staining, with findings depicted in Figure 1. Compared with the SAMR1 group, the control group exhibited noticeable alterations in myocardial tissue architecture, characterized by a disarray of cardiac cells, shrunk nucleus, uneven cytoplasmic staining, enlarged interstitial spaces, and increased fibrosis. Upon supplementation with NTs, there was an improvement in the cardiac structure: the cardiac fibers became neatly arranged; the cells were tightly arranged; the cell space was diminished, and the cytoplasm was uniformly colored. Notably, these structural enhancements were particularly pronounced in the high-dose group.

### 3.2. Effects of NTs on Antioxidant Capacity in SAMP8 Mice

**Figure 2:**
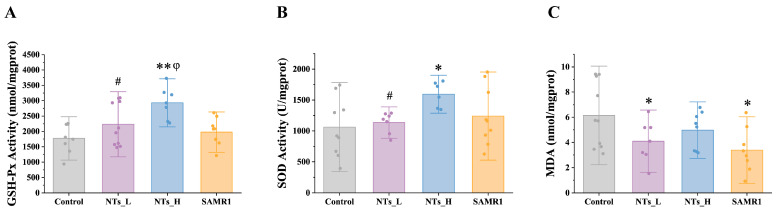
Effects of NTs on the antioxidant capacity in SAMP8 mice (*n* = 7–10 per group). (**A**) GSH-Px; (**B**) SOD; (**C**) MDA. * compared to control group, *p* < 0.05; ** compared to control group, *p* < 0.001; # compared to NTs_H group, *p* < 0.05; φ compared to SAMR1 group, *p* < 0.05.

To assess the antioxidant effects of NTs in vivo, we measured the cardiac antioxidant enzymes (SOD and GSH-Px) and the lipid peroxidation product (MDA), and the results are presented in Figure 2. Clearly, the GSH-Px activity was elevated in the NTs_H group compared to the control group (*p* < 0.001), as well as the SAMR1 and NTs_L groups (*p* < 0.05) (Figure 2A). Additionally, the SOD activity showed a significant increase in the NTs_H group than in the control and NTs_L groups (*p* < 0.05) (Figure 2B). Furthermore, the MDA levels were significantly reduced in the cardiac tissue of the NTs_L and SAMR1 groups compared to the control group (Figure 2C). These data indicated that aging augments the level of oxidative stress and diminishes the antioxidant capacity of cardiac tissue, which can be mitigated by NTs, especially at higher doses.

### 3.3. Effects of NTs on Inflammatory Markers in SAMP8 Mice

**Figure 3:**
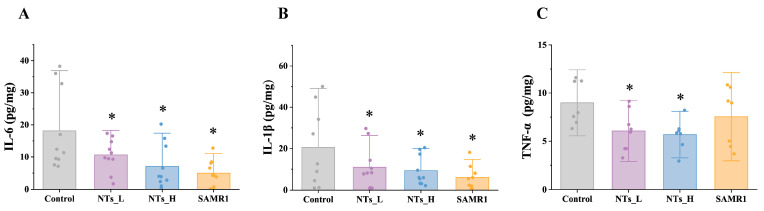
Effects of NTs on inflammatory markers in SAMP8 mice (*n* = 7–10 per group). (**A**) IL-6; (**B**) IL-1β; (**C**) TNF-α. * compared to control group, *p* < 0.05.

To explore the effects of NTs on the inflammation response in SAMP8 mice, the levels of pro-inflammatory cytokines in the cardiac tissue of mice were quantified. As depicted in Figure 3, the concentrations of IL-1β and IL-6 in the control group exhibited a significant increase compared to the SAMR1 group (*p* < 0.05), suggesting that the aging SAMP8 mice were experiencing an inflammatory state. The intervention with NTs notably reduced the concentrations of IL-6, IL-1β, and TNF-α compared to the control group (*p* < 0.05). Thus, these results indicated that NTs can mitigate age-related inflammation in the myocardium, with higher doses appearing to be more effective in the reduction of pro-inflammatory cytokines.

### 3.4. Effects of NTs on Mitochondria Function in SAMP8 Mice

**Figure 4:**
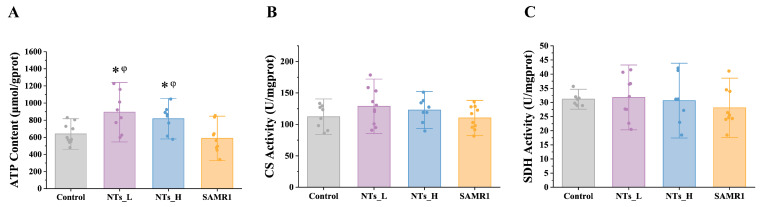
Effects of NTs on mitochondria function in SAMP8 mice (*n* = 7–10 per group). (**A**) ATP; (**B**) CS; (**C**) SDH. * compared to control group, *p* < 0.05; φ compared to SAMR1 group, *p* < 0.05.

To explore changes in the mitochondrial function within cardiac tissue, the ATP content and activities of key enzymes in energy metabolism processes (SDH and CS) were measured. As shown in Figure 4, significantly higher levels of ATP production were observed in the NT intervention groups (*p* < 0.05) compared to the control and SAMR1 groups, suggesting that NTs may enhance mitochondrial function, thereby promoting increased ATP synthesis. Furthermore, NT intervention tended to elevate the activity of CS, but no statistically significant differences were noted.

### 3.5. Effects of NTs on Cardiac Tissue Metabonomic Profiling in SAMP8 Mice

#### 3.5.1. Multivariate Analysis of Metabolic Alterations

**Figure 5:**
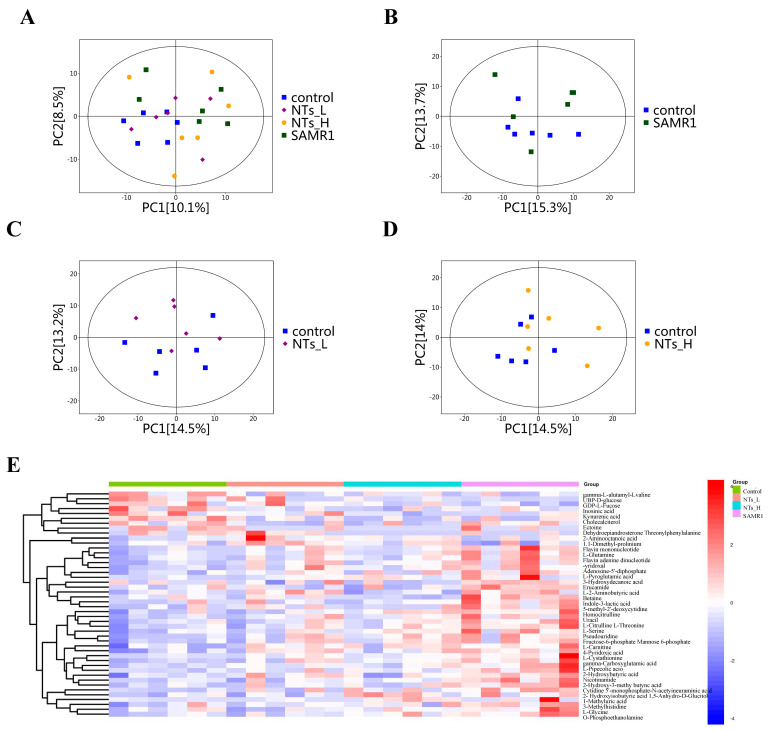
Cardiac tissue metabolomic profiling (*n* = 6 per group). (**A**) PCA score plot for the control, NTs_L, NTs_H and SAMR1 groups; (**B**) OPLS−DA score plot for the control and SAMR1 groups; (**C**) OPLS−DA score plot for the control and NTs_L groups; (**D**) OPLS−DA score plot for the control and NTs_H groups; (**E**) Heatmap of hierarchical clustering analysis for the control, NTs_L, NTs_H and SAMR1 groups.

Cardiac tissue metabolic profiles were conducted to further investigate the impact of NTs on the metabolic response in SAMP8 mice. The metabolic disturbance caused by constipation was examined through PCA and OPLS-DA. There were clear differences in the cardiac tissue metabolic profiles among different groups in the PCA score plot (Figure 5A). Furthermore, the OPLS-DA score plots showed that there was a significant separation between the control and the other three groups (Figure 5C,D), indicating that aging could alter the cardiac tissue metabolic profiles while the NT intervention could significantly improve the cardiac tissue metabolic disorder in the SAMP8 mice.

Furthermore, hierarchical cluster analysis (HCA) was applied to analyze the data across the four experimental groups. HCA is a statistical technique which categorizes data points into clusters and generates a dendrogram reflecting the distances between various elements. As depicted in Figure 5E, the analysis revealed high intra-group correlation and no inter-group crossover in the four groups of mice, indicating that the NT intervention altered the cardiac tissue metabolites of the mice.

#### 3.5.2. Metabolic Alterations Between the Control and the SAMR1 Groups

**Figure 6:**
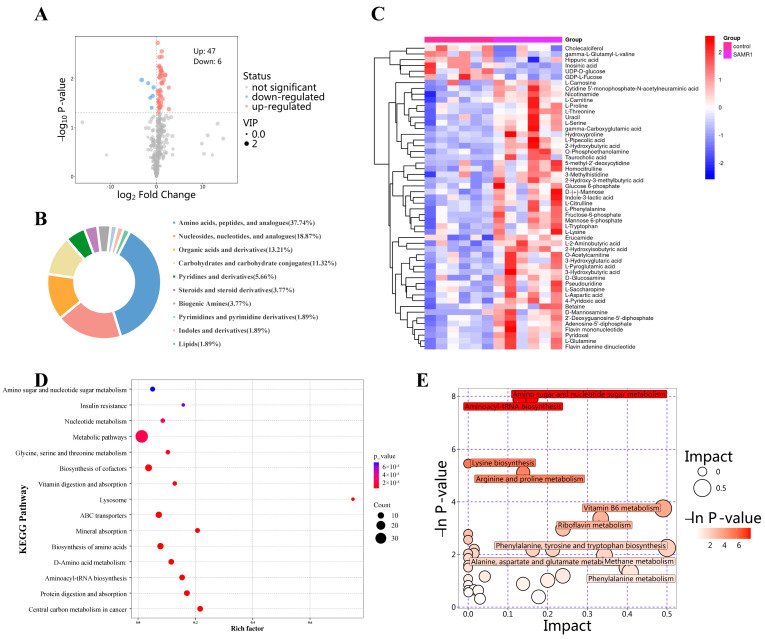
Metabolic alterations between the control and SAMR1 groups (*n* = 6 per group). (**A**) Volcano plot of DEMs; (**B**) Classification of DEMs; (**C**) Heatmap of DEMs; (**D**) KEGG enrichment analysis; (**E**) Bubble plot of KEGG pathway analysis.

A total of 53 DEMs in the cardiac tissue between the control and the SAMR1 groups were detected, of which 47 metabolites were upregulated while six metabolites were downregulated in the SAMR1 group compared to the control group (Figure 6A,C). Figure 6B showed the classification of the DEMs, and most of the metabolites were defined as amino acids and nucleosides, nucleotides, and their analogues. Metabolic pathway analysis and enrichment analysis were performed to identify the pathways related to DEMs. The enrichment analysis showed the highly affected metabolite sets, and the top 15 metabolite sets and the number of metabolites were presented in Figure 6D. Metabolic pathway analysis showed that amino sugar and nucleotide sugar metabolism, aminoacyl-tRNA biosynthesis, lysine biosynthesis, arginine and proline metabolism, and vitamin B6 metabolism and riboflavin metabolism changed most significantly between the control and the SAMR1 groups (Figure 6E). The changes in these pathways that are linked to energy metabolism suggest alterations in cardiac energy metabolism in aging mice.

#### 3.5.3. Metabolic Alterations Between the Control and the NTs_L Groups

**Figure 7:**
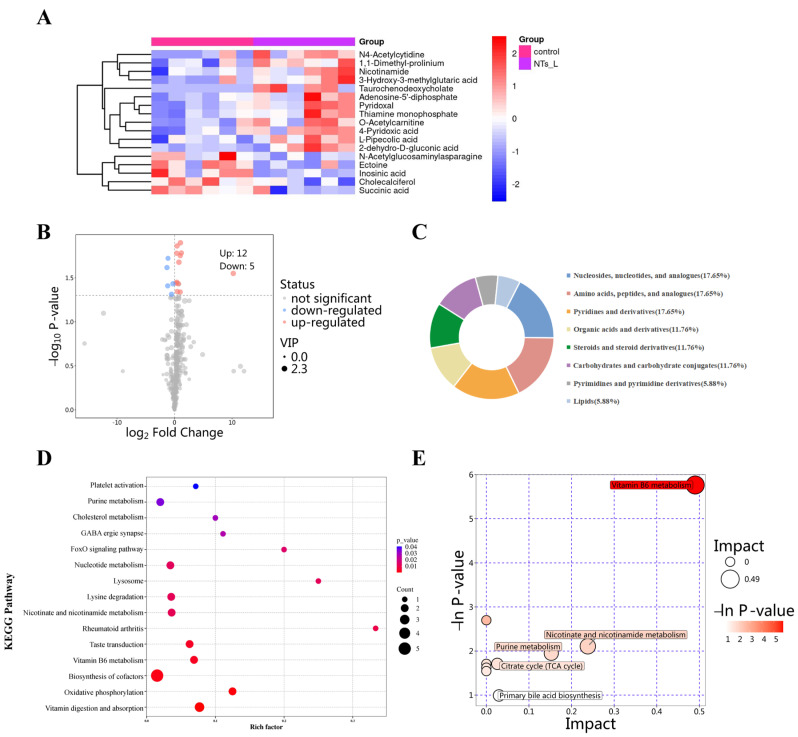
Metabolic alterations between the control and NTs_L groups (*n* = 6 per group). (**A**) Heatmap of DEMs; (**B**) Volcano plot of DEMs; (**C**) Classification of DEMs; (**D**) KEGG enrichment analysis; (**E**) Bubble plot of KEGG pathway analysis.

Overall, 17 DEMs between the control and the NTs_L groups were identified, of which 12 metabolites were significantly upregulated and five metabolites were significantly downregulated in the NTs_L group, as compared with the control group (Figure 7A,B). According to the classification, most of the metabolites were defined as the nucleosides and analogues group, amino acids and analogues group, and pyridines and derivatives group (Figure 7C). Enrichment analysis showed that DEMs were enriched to 15 metabolite sets such as purine metabolism, nucleotide metabolism, lysine degradation, nicotinate and nicotinamide metabolism, biosynthesis of cofactors, oxidative phosphorylation, and vitamin digestion and absorption (Figure 7D). The pathway analysis also corroborated the results of the enrichment analysis. As shown in Figure 7E, the most significant pathways were related to vitamin B6 metabolism, nicotinate and nicotinamide metabolism, and purine metabolism and the citrate cycle, which indicated that the low-dose NTs can improve cardiac energy metabolism via the related cofactor pathways.

#### 3.5.4. Metabolic Alterations Between the Control and the NTs_H Groups

**Figure 8:**
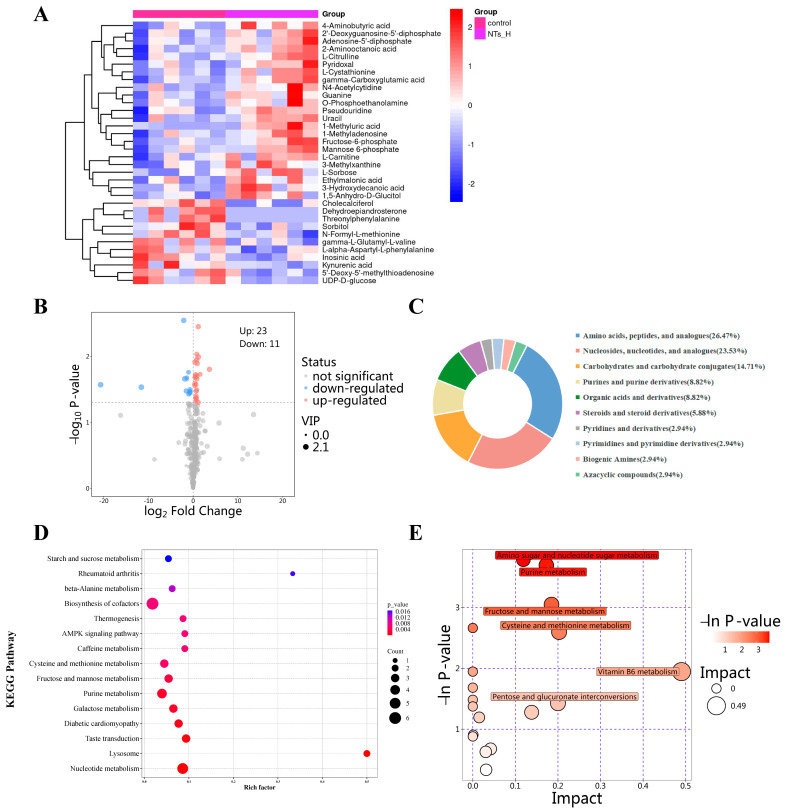
Metabolic alterations between the control and NTs_H groups (*n* = 6 per group). (**A**) Heatmap of DEMs; (**B**) Volcano plot of DEMs; (**C**) Classification of DEMs; (**D**) KEGG enrichment analysis; (**E**) Bubble plot of KEGG pathway analysis.

Between the control and the NTs_H groups, a total of 34 DEMs were identified, of which 23 metabolites were significantly upregulated and 11 metabolites were significantly downregulated in the NTs_H group compared to the control group (Figure 8A,B). A large proportion of these DEMs belonged to the amino acids and analogues group, nucleosides and analogues group, and carbohydrates and carbohydrate conjugates group, which was similar to the changes between the control and SAMR1 groups (Figure 8C). Enrichment analysis showed that DEMs were enriched to 15 metabolite sets such as biosynthesis of cofactors, purine metabolism, nucleotide metabolism, and AMPK signaling pathway (Figure 8D). Metabolic pathway analysis showed that amino sugar and nucleotide sugar metabolism, purine metabolism, fructose and mannose metabolism, cysteine and methionine metabolism, vitamin B6 metabolism and pentose and glucuronate interconversions changed most significantly between the control and the NTs_H groups (Figure 8E). The results between these two groups have a good consistency with those between the Control and SAMR1 group, suggesting that the NT intervention reversed the changes in cardiac metabolome caused by aging.

### 3.6. Effect of NTs on Mitochondria Biogenesis in SAMP8 Mice

**Figure 9:**
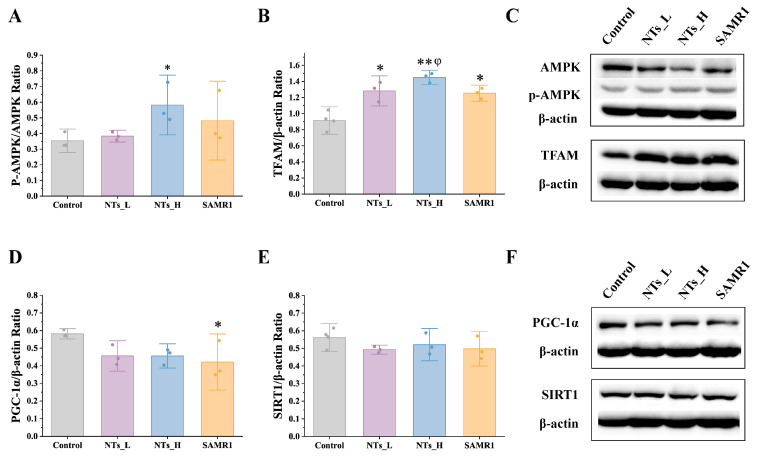
Effects of NTs on the protein expressions of AMPK, TFAM, PGC-1α, and SIRT1 in SAMP8 mice (*n* = 3–4 per group). (**A**) Western blot analysis of p-AMPK/AMPK; (**B**) Western blot analysis of TFAM; (**C**) Western blot analysis of p-AMPK, AM,PK and TFAM; (**D**) Western blot analysis of PGC-1α; (**E**) Western blot analysis of SIRT1; (**F**) Western blot analysis of PGC-1α, and SIRT1. * compared to control group, *p* < 0.05; ** compared to control group, *p* < 0.001; φ compared to SAMR1 group, *p* < 0.05.

As depicted in Figure 9, the NTs_H group exhibited a higher expression of AMPK phosphorylation levels compared to the control group (*p* < 0.05), indicating that the high dose of NTs may activate the AMPK pathway. Furthermore, the TFAM expression was upregulated in the NTs_H (*p* < 0.001) and NTs_L and SAMR1 groups (*p* < 0.05) compared to the control group. However, no statistical difference was observed in the expression of PGC-1α and SIRT1 proteins.

### 3.7. Effect of NTs on Cardiac Glycolysis in SAMP8 Mice

**Figure 10:**
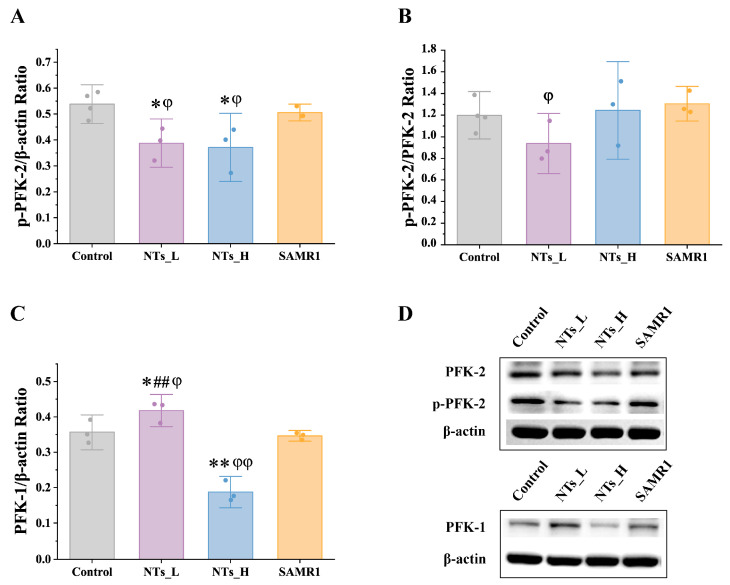
Effects of NTs on the protein expressions of PFK-2 and PFK-1 in SAMP8 mice (*n* = 3–4 per group). (**A**) Western blot analysis of p- PFK-2; (**B**) Western blot analysis of p- PFK-2/PFK-2; (**C**) Western blot analysis of PFK-1; (**D**) Western blot analysis of p- PFK-2, PFK-2 and and PFK-1. * compared to control group, *p* < 0.05, ** compared to control group, *p* < 0.001; ## compared to NTs_H group, *p* < 0001; φ compared to SAMR1 group, *p* < 0.05; φφ compared to SAMR1 group, *p* < 0.001.

As shown in Figure 10, in comparison to the control group, PFK-2 expression was downregulated in the NTs_H and SAMR1 groups, whereas no statistical difference of the PFK-2 phosphorylation levels was observed between the control group and NTs_H as well as SAMR1 groups. For PFK-1, high-dose and low-dose NT interventions showed opposite effects. Compared with the control group, NTs_L upregulated (*p* < 0.05) while NTs_H downregulated (*p* < 0.001) the expression of PFK-1.

## 4. Discussion

This significant study investigated the effects of long-term exogenous NT treatment on cardiac aging in SAMP8 mice and initially explored the possible regulatory mechanisms. As the cardiac structure and function underwent age-related alterations, we observed signs of cardiac aging in 12-month-old SAMP8 mice, which were effectively attenuated by nucleotide treatment. The SAMP8 mouse model is widely used in age-related studies [26] and regarded as a suitable model for evaluating cardiac aging [24]. Aging may contribute to an increase in chronic inflammation, apoptosis, and necrosis within cardiac tissue, which in turn leads to an enlarged muscle cell volume, collagen deposition, fibrosis, and ultimately, a significant alteration in cardiac structure [27]. In this study, we noted that increasing age led to significant cardiac morphological changes in mice, including a disarray of cardiac cells, enlargement of the interstitial spaces, shrunk nucleus, uneven cytoplasmic staining, and increased cardiac fibrosis. These changes were markedly mitigated by the intervention of NTs. Changes in the structure of the cardiac tissue can ultimately affect its function. Elevated cardiac fibrosis contributes to cardiac dysfunction, typified by either diastolic or systolic failure [28,29]. Our findings suggest that sustained NT supplementation could retard the advancement of cardiac aging.

A wealth of epidemiological studies supported that inflammation, akin to aging, is a critical risk factor for cardiovascular disease. Studies has illuminated that damage to cardiac muscle tissue is coupled with elevated levels of pro-inflammatory factors [30,31]. Recent studies have put forward the concept of “inflamm-aging”, emphasizing low-grade inflammation observed in the elderly that contributes to the risk of CVD [32]. In alignment with these findings, our study identified an increased inflammatory response in the cardiac tissue of aged mice compared to the control group. Importantly, NT intervention notably diminished the levels of inflammatory factors, including IL-6, IL-1β, and TNF-α. This suggests that NTs may play a protective role in maintaining the structural and functional integrity of the heart by regulating the levels of pro-inflammatory cytokines, thereby decelerating the aging process of the heart. Our findings corroborate previous studies that have underscored the capacity of NTs to mitigate age-related conditions through the modulation of inflammatory pathways [22].

A primary contributor to age-related cardiac dysfunction is the metabolic remodeling of cardiomyocytes, which is marked by a decline in fatty acid oxidation rates, an elevation in glycolysis, and a loss of metabolic flexibility [33]. Utilizing metabolomics technology, this investigation delved into the metabolic changes in aging cardiac tissue. The findings indicated that the SAMR1 group exhibited pronounced metabolic alterations in contrast to the control group, identifying 53 DEMs. Key energy metabolic pathways were disrupted by aging, including amino sugar and nucleotide sugar metabolism, aminoacyl-tRNA biosynthesis, lysine biosynthesis, arginine and proline metabolism, vitamin B6 metabolism, and riboflavin metabolism. Overall, the metabolic profiles in the high-dose NT intervention group mirrored those of the SAMR1 group, indicating that the 1.2 g/kg NT intervention can ameliorate the metabolic derangements of cardiac tissue caused by aging. This study found that aging led to a marked reduction in fructose-6-phosphate levels, which was restored following high-dose NT intervention (Supplementary Figure S1). Fructose-6-phosphate is crucial for glycolysis, the metabolic pathway that converts glucose to pyruvate and generates ATP under both aerobic and anaerobic conditions. In conditions such as myocardial infarction, ischemia, and heart failure, glycolysis is enhanced to increase energy supply [34,35,36]. However, glycolysis has also been implicated in the activation of cardiac fibrosis [37]. PFK-2 is a key glycolytic enzyme that facilitates the conversion of fructose-6-phosphate to 1,6-fructose diphosphate [38]. Western blotting analysis showed that the expression of PFK-2 protein in the NT intervention group was significantly reduced compared to the control group, with a similar reduction observed for PFK-1 protein in the high-dose NT group. These changes in protein expression were consistent with the metabolomic data. The diminished glycolytic substrates and elevated glycolytic-related enzymes in the control group suggest that aging boosts myocardial glycolytic activity, a condition that the NT intervention group, especially the high-dose group, appears to rectify by enhancing cellular energy metabolism.

Additionally, metabolic profiling also revealed other intriguing observations. L-Citrulline was significantly upregulated in both the SAMR1 and NTs_H groups as compared to the control group. Recent studies have found that L-Citrulline might offer CVD benefits by modulating blood pressure and endothelial function [39,40,41,42]. L-Carnitine, known for its cardioprotective properties through the modulation of energy metabolism and fatty acid oxidation, exhibited elevated levels following the SAMR1 and NTs_H interventions [43]. The Bogalusa Heart Study, leveraging metabolomics, identified sixteen novel metabolites including the gamma-L-Glutamyl-L-valine as independent of traditional risk factors associated with arterial stiffness [44]. In our study, this specific metabolite exhibited an age-related increase, yet its level was downregulated by the high-dose NT supplementation. The NT intervention, enhancing the beneficial metabolites and downregulating the risk metabolites, indicated that high-dose NTs improved cardiac metabolism and exhibited heart benefits. Furthermore, inosinic acid (IMP) levels were downregulated in the other three groups relative to the control group. Purine nucleotide synthesis occurs via de novo and salvage pathways, with IMP being crucial for the former [45]. The higher content of IMP in the control group suggested an upregulation of de novo synthesis to accommodate nucleotide demand during aging. The supplementation of high-dose NTs, which reduced IMP levels, implied that the intake of nucleotides directly supplies salvage synthesis substrates, thus reducing reliance on de novo synthesis. Given the intricate nature of nucleotide synthesis, involving multiple metabolites and enzymes, further studies are warranted.

Numerous studies have established a link between oxidative stress, mitochondrial function, and cardiac aging [46]. Oxidative stress occurs when there is an overproduction of ROS without adequate antioxidant defenses. Evidence supporting the detrimental role of mitochondria-derived ROS in cardiac aging is provided by studies showing that mitochondrial catalase overexpression slows this aging process [47]. Studies have shown that NTs can enhance the antioxidant capacity in various tissues of aging mice, including the skin and testicles [22,25,48]. In the current study, the low-dose NT treatment significantly reduced the content of MDA; both of the two doses of NTs increased the activity of key antioxidant enzymes GSH-Px, and the high-dose NTs increased the activity of another antioxidant enzymes SOD, compared to the control group. These findings suggest that the antioxidant effects of NTs also apply to cardiac tissue, and their intervention can effectively reduce the level of oxidative stress in the heart, potentially delaying the aging of cardiac tissue.

Mitochondria play a crucial role in cardiac tissue, serving as the primary source of energy through oxidative phosphorylation (OXPHOS) and meeting 90% of the heart’s ATP demands [49]. Beyond bioenergetics, they also serve as a metabolic hub that regulates the balance between glucose metabolism and fatty acid oxidation. However, with aging, there is a noticeable decline in both the quantity and integrity of mitochondria within cardiomyocytes [49]. Studies have shown that, although the mtDNA copy number and cardiac CS activity and content, which are important indicators of mitochondrial function, are decreased in aged skeletal muscle and liver compared with adults, these parameters remain unchanged in the aged heart [50,51]. Consistent with previous findings, our study did not find statistical differences in the CS activity as well as the SDH activity between the SAMR1 and SAMP8 mice. Nevertheless, we observed that the NT intervention increased ATP content in cardiac tissue, implying a regulatory effect on mitochondrial energy metabolism. Besides impacting energy metabolism, the persistent presence of defective mitochondria in the aging heart also results in the production of oxidants and enhanced oxidative damage, as well as the activation of oxidative signaling that leads to cell death [10]. The mechanism behind cardiac aging caused by mitochondrial dysfunction likely involves impaired mitochondrial biogenesis [52]. We concentrated on the AMPK pathway to elucidate the underlying mechanism. AMPK, a cellular energy sensor activated during energy stress, plays a key role in maintaining energy homeostasis and regulates processes such as mitochondrial biogenesis, redox regulation, cell growth and proliferation, and cellular inflammation. Recent studies have demonstrated that AMPK has protective effects on cardiovascular function, encompassing systolic, antioxidant, anti-inflammatory, and anti-atherosclerosis benefits [53]. TFAM, a transcription factor that maintains mtDNA stabilization and initiates mtDNA replication, is essential for mitochondrial biogenesis. Our study indicated that the NT intervention activated the AMPK protein and increased TFAM protein expression, suggesting that TFAM is regulated by the AMPK pathway. Studies have shown that AMPK and SIRT1 co-activate PGC-1α [30,54,55], and PGC-1α can directly influence TFAM to regulate mitochondrial function [19]. However, we did not find significant differences in the SIRT1 and PGC-1α protein expressions, possibly due to the lower sensitivity of protein expression assays compared to gene expression analysis. A study related to heart failure reported downregulation of PGC-1α target genes despite increased PGC-1α expression [56]. Our findings also suggested that alternative pathways may be involved in AMPK-mediated TFAM activation.

In addition, the dosage is a noticeable factor in this study. Taken together, the higher dosages of NTs (1.2 g/kg) exert more positive effects across multiple indicators than the low-dose group (0.3 g/kg). Notably, the high-dose NTs treatment led to significant improvement in metabolomic profiles, antioxidant enzyme activities, and the expression levels of proteins involved in mitochondrial biogenesis. A previous study also found that high-dose NTs improved the mitochondrial function, which is consistent with our findings [22]. This suggests that the beneficial effect of NTs on cardiac tissue is dose-dependent, and the underlying mechanism may be related to oxidative stress and energy metabolism. However, this study did not focus on dose-related analyses, leaving the optimal dosage for cardiac aging undetermined. Further exploration is required to elucidate the specific mechanisms. Overall, the findings highlight the potential advantages of high doses of NTs and underscore the importance of further research to refine cardiac aging treatment strategies.

This study provides preliminary insights into the effects of NTs on cardiac tissue and underscores the complexity of oxidative stress, inflammation, mitochondrial function, and energetic metabolism in aging cardiac tissue. Further research is essential to elucidate and validate the specific mechanisms by which NT supplementation influences cardiac aging. A limitation of this study is the less robust than expected link between NT intervention and anti-aging benefits on cardiac tissue. Notably, while significant differences were observed in metabolomics, MDA levels, inflammatory biomarkers (IL-1β and IL-6), and TFAM protein expression between the control and SAMR1 groups, other outcomes lacked statistical significance, possibly due to varying sensitivities of indices to cardiac aging. This interesting phenomenon merits future investigation. Additionally, the absence of continuous cardiac function monitoring in mice during the intervention limits our ability to correlate molecular changes with functional outcomes. Future studies will incorporate such monitoring to better assess the effects of NTs on cardiac function and explore their mechanisms in cardiac health, potentially leading to new strategies for preventing and managing cardiac aging.

## 5. Conclusions

Together, our findings suggest that exogenous NTs exert beneficial effects on the cardiac tissues of SAMP8 mice, potentially mitigating the cardiac aging process. Our study provides evidence that NTs ameliorate age-related histopathological changes, improve antioxidant capacity, and attenuate inflammatory responses in SAMP8 mice. Furthermore, the NTs intervention improved energy metabolism in cardiac tissue, which may be related to the activation of the AMPK pathway, improved mitochondrial biogenesis, and upregulated TFAM.

## Figures and Tables

**Table 1:** Animals and Treatment.

Groups	Feed	Number of Animals	Survival Animal Numbers
Control	Standard Feed	15	12
NTs_L	Standard Feed + 0.3 g/kg NTs	15	14
NTs_H	Standard Feed + 1.2 g/kg NTs	15	12
SAMR1	Standard Feed	15	12

## Data Availability

Data will be made available on request.

## Supplementary Materials

The following are available online at https://www.mdpi.com/article/10.3390/nu16223851/s1, Figure S1: Quantification of Fructose-6-phosphate by metabolomics.

## Abbreviations

AMP	5′-adenosine monophosphate
AMPK	AMP-activated protein kinase
CMP	5′-cytimidine monophosphate
Control	normal control group
CS	citrate synthase
CVD	cardiovascular disease
DEMs	differentially expressed metabolites
GMP	5′-guanosine monophosphate
GSH-Px	glutathione peroxidase
HE	hematoxylin-eosin
IL-1β	interleukin-1 β
IL-6	interleukin-6
IMP	inosinic acid
MDA	malondialdehyde
NTs	nucleotides
NTs_H	high-dose NTs group
NTs_L	low-dose NTs group
OPLS-DA	orthogonal projections to latent structures-discriminate analysis
OXPHOS	oxidative phosphorylation
PCA	principal component analysis
PGC-1α	peroxisome proliferator-activated receptor-gamma coactivator-1α
ROS	reactive oxygen species
SAMP8	senescence-accelerated mouse prone-8
SAMR1	senescence-accelerated mouse resistant 1
SDH	succinate dehydrogenase enzyme
SOD	superoxide dismutase
SPF	specific-pathogen free
TFAM	mitochondrial transcription factor A
TNF-α	tumor necrosis factor-α
UMP	5′-disodium uridine monophosphate
VIP	variable importance in the projection
