# Supplementary material for: Exogenous Nucleotides Mitigate Cardiac Aging in SAMP8 Mice by Modulating Energy Metabolism Through AMPK Pathway
Source: Nutrients. 2024 Nov 11;16(22):3851. doi: 10.3390/nu16223851 (PMC11597617; doi:10.3390/nu16223851)
Supplement: Supplementary file 1 [file nutrients-16-03851-s001.zip › nutrients-3281676-supplementary.pdf]

SUPPLEMENTARY FIGURES

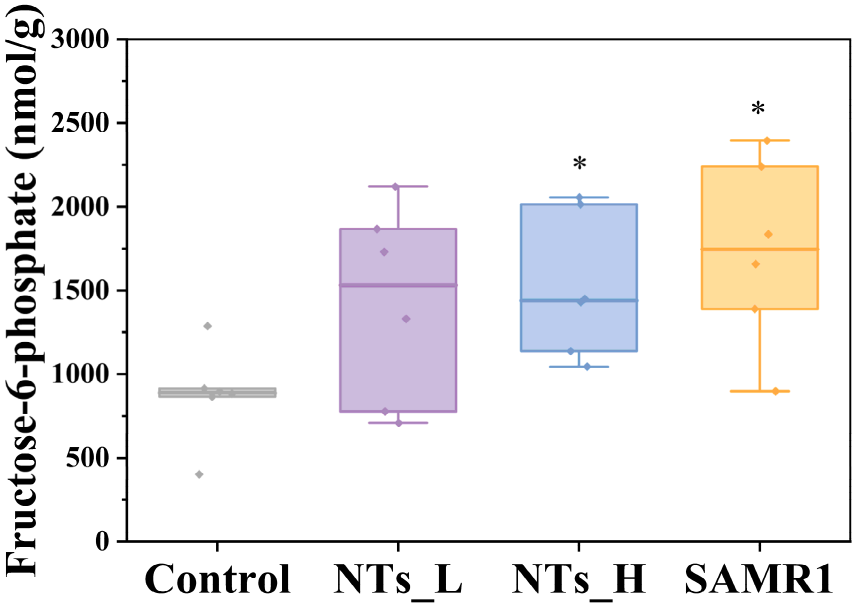

Figure S1 Quantification of Fructose-6-phosphate by metabolomics. * Compared to Control group $p < 0.05$.
